# Computationally-guided design and selection of high performing ribosomal active site mutants

**DOI:** 10.1093/nar/gkac1036

**Published:** 2022-12-09

**Authors:** Camila Kofman, Andrew M Watkins, Do Soon Kim, Jessica A Willi, Alexandra C Wooldredge, Ashty S Karim, Rhiju Das, Michael C Jewett

**Affiliations:** Department of Chemical and Biological Engineering, Northwestern University, Evanston, IL 60208, USA; Chemistry of Life Processes Institute, Northwestern University, Evanston, IL 60208, USA; Center for Synthetic Biology, Northwestern University, Evanston, IL 60208, USA; Department of Biochemistry, Stanford University, Stanford, CA 94305, USA; Prescient Design, Genentech, South San Francisco, CA 94080, USA; Department of Chemical and Biological Engineering, Northwestern University, Evanston, IL 60208, USA; Chemistry of Life Processes Institute, Northwestern University, Evanston, IL 60208, USA; Center for Synthetic Biology, Northwestern University, Evanston, IL 60208, USA; Inceptive Nucleics, Inc., Palo Alto, CA 94304, USA; Chemistry of Life Processes Institute, Northwestern University, Evanston, IL 60208, USA; Center for Synthetic Biology, Northwestern University, Evanston, IL 60208, USA; Chemistry of Life Processes Institute, Northwestern University, Evanston, IL 60208, USA; Center for Synthetic Biology, Northwestern University, Evanston, IL 60208, USA; Department of Chemical and Biological Engineering, Northwestern University, Evanston, IL 60208, USA; Chemistry of Life Processes Institute, Northwestern University, Evanston, IL 60208, USA; Center for Synthetic Biology, Northwestern University, Evanston, IL 60208, USA; Department of Biochemistry, Stanford University, Stanford, CA 94305, USA; Department of Physics, Stanford University, Stanford, CA 94305, USA; Department of Chemical and Biological Engineering, Northwestern University, Evanston, IL 60208, USA; Chemistry of Life Processes Institute, Northwestern University, Evanston, IL 60208, USA; Center for Synthetic Biology, Northwestern University, Evanston, IL 60208, USA; Robert H. Lurie Comprehensive Cancer Center and Simpson Querrey Institute, Northwestern University, Chicago, IL 60611, USA

## Abstract

Understanding how modifications to the ribosome affect function has implications for studying ribosome biogenesis, building minimal cells, and repurposing ribosomes for synthetic biology. However, efforts to design sequence-modified ribosomes have been limited because point mutations in the ribosomal RNA (rRNA), especially in the catalytic active site (peptidyl transferase center; PTC), are often functionally detrimental. Moreover, methods for directed evolution of rRNA are constrained by practical considerations (e.g. library size). Here, to address these limitations, we developed a computational rRNA design approach for screening guided libraries of mutant ribosomes. Our method includes *in silico* library design and selection using a Rosetta stepwise Monte Carlo method (SWM), library construction and *in vitro* testing of combined ribosomal assembly and translation activity, and functional characterization *in vivo*. As a model, we apply our method to making modified ribosomes with mutant PTCs. We engineer ribosomes with as many as 30 mutations in their PTCs, highlighting previously unidentified epistatic interactions, and show that SWM helps identify sequences with beneficial phenotypes as compared to random library sequences. We further demonstrate that some variants improve cell growth *in vivo*, relative to wild type ribosomes. We anticipate that SWM design and selection may serve as a powerful tool for rRNA engineering.

## INTRODUCTION

The ribosome is a complex macromolecular machine that has evolved to synthesize proteins by catalyzing peptide bonds between amino acids. Essential for all life, the ribosome is considered to be a ribozyme, as its catalytic active site, the peptidyl transferase center (PTC), is primarily composed of ribosomal RNA (rRNA) (1–4). Consequently, ribosome assembly and function are tightly linked to rRNA folding and stability, and the ribosome's sequence has been evolutionarily constrained to enable folding into a structure capable of catalyzing peptide bonds rapidly and reliably (5,6).

The production of modified ribosomes with biochemical defects (7–10) or altered capabilities (e.g. β-amino acid incorporation (11,12)) serves as an important tool to better understand molecular translation and enable synthetic biology applications (13). However, designing and engineering mutant ribosomes is far from a trivial undertaking because ribosomal mutations can disrupt translation in ways that are often lethal to cells. For example, previous efforts to create modified ribosomes have shown that making even single point mutations to the PTC sequence can nullify the ribosome's ability to properly assemble or catalyze bond formation (14–17). Not only is it difficult to identify functional small-scale mutations, but some detrimental mutations can also be rescued by synergistic mutations in adjacent or distal regions, highlighting the design challenges of rRNA engineering and importance of considering epistatic relationships between residues when designing libraries (18,19).

While tethered ribosomes (20–23) or cell-free strategies (24–28) can be used to identify functionally detrimental ribosomal mutations, efforts to build modified ribosomes remain hampered by practical considerations in making and evaluating rRNA libraries. For example, the combinatorial space for rRNA evolution is large, such that random mutagenesis and selection approaches cannot be feasibly used to screen all possible variants. In addition, due to primer bias, randomized libraries constructed using PCR are known to have imbalanced initial populations, skewing assessments of a library's members by overemphasizing the more common ones (29). PCR-based library construction approaches are also difficult to apply to multiple regions of rRNA that are close in three-dimensional space but not primary sequence space, which is common in the structurally complex PTC (30). A further challenge is that DNA libraries are typically propagated in cells, where transformation idiosyncrasies limit library size (11,31–33). Alternative approaches are thus needed to test unbiased rRNA libraries of larger size and complexity such that we can explore diverse energy landscapes via large-scale sequence changes and identify mutant ribosomes with significantly modified architectures.

Here, to explore rRNA design rules with high-throughput methods for identifying synergistic mutations, we develop a computationally guided approach for making modified ribosomes. First, we use a stepwise Monte Carlo method (SWM) in Rosetta to score, rank, and select rRNA library members using an all-atom energy score (34). While previous methods have used computationally expensive, low-resolution coarse graining or small perturbations to fully build conformations (35), SWM requires much less computational power to reach an equivalent level of atomic accuracy (34). This approach also allows us to define libraries in three-dimensional space, including any residues that are potentially interacting and could mutate to play compensatory roles. We combine this computational approach with a high throughput *in vitro* ribosome synthesis, assembly, and translation (iSAT) screening platform (13,36–38) to test the computationally identified mutants, allowing us to rapidly assay promising candidates. The resulting ribosome mutants highlight the flexibility of the PTC to large-scale mutations and elucidate previously unknown epistatic relationships between distal regions of the PTC. Unexpectedly, many of these highly mutated variants can support life in cells with only minor phenotypic effects. We anticipate that our high-throughput, computationally guided approach will allow for improved studies of complex rRNA libraries to ultimately enable novel ribosomal activity as well as deeper understanding of molecular translation.

## MATERIALS AND METHODS

### SWM design simulations

To obtain an initial structure for stepwise Monte Carlo design, a crystal structure of the *Escherichia coli* ribosome (PDB code: 4YBB (39)) was obtained and loaded into PyMOL. The residues of interest, local to a particular site in the ribosome, were selected, and that selection was expanded to include a 25.0 Å sphere of neighboring residues, enough to encompass several shells of indirect interactions. The full selection, including both residues of interest and neighbors, were saved to a ‘native’ PDB file. This ‘native’ file was passed to a Python script distributed with the Rosetta application, *tools/rna_tools/pdb_util/pdb2fasta.py*, to obtain a corresponding FASTA-formatted file with appropriate numbering. RNA sequences were defined in lowercase font for the input and output text; therefore, we have used lowercase to display mutated RNA sequences in this work, while referencing specific point mutants in uppercase in the text. Finally, the 25.0 Å sphere of neighbors, but omitting the actual residues of interest, were saved to a ‘starting’ PDB file, ready for stepwise Monte Carlo design.

The sequence positions within the FASTA file that corresponded to the residues of interest were edited with a text editor to ensure the design simulation would sample any nucleotide but the wild-type nucleic acid identity: *a* was changed to *b* (the IUPAC ambiguous single-letter code representing ‘anything but adenosine’); *c* was changed to *d*; *g* was changed to *h*; *u* was changed to *v*. Because the region being redesigned would be free to resample its backbone conformation, some ‘adaptation’ between the fully flexible designed region and the totally rigid crystal context was necessary. To this end, the residues adjacent in primary sequence to any redesigned residue were indicated to the *-extra_min_res* flag: these residues, although not subject to explicit backbone sampling, were subject to quasi-Newtonian energy minimization along with the designed residues during simulation.

Simulations for rRNA helices 73, 75 and 91 were run for 1 000 Monte Carlo cycles, while simulations for helix 92 were run for 2 000 Monte Carlo cycles due to its structural complexity. At least 10 000 independent trajectories were run for each library. Full code examples for setting up, conducting, and analyzing design simulations are provided at https://doi.org/10.5281/zenodo.7230453; documentation for stepwise Monte Carlo that includes details on design simulations is available at https://new.rosettacommons.org/docs/latest/application_documentation/stepwise/stepwise_monte_carlo/stepwise.

### Sequence alignment and analysis

A dataset consisting of 1 614 pre-aligned and phylogenetically arranged bacterial and archaeal 23S sequences was downloaded and analyzed as previously published (15). Full code examples of the analyses are provided at https://doi.org/10.5281/zenodo.7230166.

### Forward folding with SWM

In a design simulation, different sequences may be the lowest scoring frame of a trajectory at significantly variable frequencies. As a result, it can be difficult to make confident comparisons between the best energy sampled for two sequences or to set a strict threshold to select a small number of desired variants. Instead of establishing a strict cutoff selecting only a few variants for experimental characterization based on variable quantities of data, we ran individual ‘forward-folding’ simulations on a larger number of fixed sequences, using the 200 top-scoring sequences from the design simulation. These ‘forward-folding’ simulations used only 500 cycles and generated exactly 400 models each, ensuring an ‘apples to apples’ comparison among sequences for the final selection that would be inaccessible to a design simulation alone. We ran these simulations specifically for helix 75, where we were interested in whether lower scores would correlate to superior performance in iSAT, so accuracy and fair sampling for the single highest score was paramount. We elected not to repeat the simulations for the other helices due to computational resource constraints. Full code examples for setting up, conducting, and analyzing forward folding simulations are provided at https://doi.org/10.5281/zenodo.7230453.

### Plasmid construction & preparation

Plasmids were ordered from Twist in two backbones: one in the pT7rrnB backbone (37) and one in pAM552G (22). Plasmids used for testing of variants in iSAT were built using pT7rrnB, a 7 311-bp plasmid. This plasmid carries an *Escherichia coli* rRNA operon, rrnB, under the control of the T7 promoter, as well as the ampicillin resistance gene. Constructs from Twist in the pT7rrnB backbone were transformed into chemically competent *E. coli* Dh10β cells and plated on LB plates supplemented with 50 μg/ml Carbenicillin (Cb_50_). Plates were incubated at 37°C overnight. Single colonies were picked and grown overnight at 37°C in 50-ml of LB media supplemented with 100 μg/ml Carbenicillin (Cb_100_). Plasmids were then purified using the ZymoPure II Plasmid Miniprep Kit. The resulting plasmids were further purified via ethanol precipitation using 5 M NH_4_OAc for use in cell-free reactions.

Plasmids used for testing of variants in the “Squires” strain (SQ171fg) (40,41) were built using the 7 451-bp pAM552G plasmid in POP2136 cells. Like the pT7rrnB plasmid, pAM552G carries a copy of the rrnB operon as well as an ampicillin resistance gene. However, in pAM552G, expression of rrnB is under control of the phage lambda pL promoter, which is in turn regulated by the temperature-sensitive bacteriophage lambda cI857 repressor (42). Plasmids from Twist were transformed into chemically competent *E. coli* POP2136 cells and plated on LB plates containing Cb_50_. Plates were incubated at 30°C to prevent expression of rRNA from the plasmid. Colonies were picked and grown overnight in 5-ml Cb_100_ cultures and grown at 30°C to continue repression of rRNA expression. Plasmids were purified using the ZymoPURE Plasmid Miniprep Kit. Purified plasmids were then transformed into electrocompetent SQ171fg cells (22,40). Of note, both plasmids contain an A2058G mutation, which endows the resulting ribosome with Erythromycin (Ery) resistance. Ery is used for the *in vivo* selection.

### Strain harvest and extract preparation

S150 lysates, total protein of the 70S ribosome (TP70) and T7 RNA Polymerase were prepared as previously reported (25,37). 10 ml of an overnight culture of *E. coli* (MRE600 strain) cells were added into a liter of 2× YTPG medium (2xYTP with 18 g/l of glucose) and grown at 37°C with shaking at 250 rpm until OD600 reached 3. Culture was spun down at 5 000 × *g* for 10 min and kept on ice between all transfer steps. Supernatant was removed and pellet was resuspended in S30 buffer (10 mM TrisOAc pH 8.2, 14 mM Mg(OAc)_2_, 60 mM KOAc). Cell suspension was spun at 10 000 × *g* for 3 min twice more, removing supernatant between each spin and resuspending in 40 ml of fresh S30 buffer. After the third spin, cell pellets were weighed and flash frozen with liquid nitrogen before storing at –80°C. S30 Buffer was then added at a ratio of 5 ml per 1 g of cell mass, and cells resuspended by vortexing until fully thawed. 100 μl of HALT Protease Inhibitor Cocktail was added per 10 ml cell suspension, and 75 μl of Takara Recombinant RNase Inhibitor was added per 4 g of dry cell mass. Cells were lysed at ∼25 000 psi with a C3 Avestin Homogenizer and a second aliquot of Takara Recombinant RNase Inhibitor was added at a ratio of 75 μl per 4 g of initial pellet. Cell debris were pelleted by centrifugation at 12 000 × *g* at 4°C for 15 min. Supernatant (S12 extract) was recovered for S150 extract preparation and layered on top of an equivalent volume of sucrose cushion buffer (20 mM Tris–HCl (pH 7.2 at 4°C), 100 mM NH_4_Cl, 10 mM MgCl_2_, 0.5 mM EDTA, 2 mM DTT, 37.7% sucrose) in Ti70 tubes. Samples were then ultracentrifuged at 90 000 × g for 18 h, after which the supernatant was transferred into fresh Ti70 tubes and spun at 150 000 × g for 3 h and pellets were gently washed with buffer C (10 mM Tris–OAc (pH 7.5 at 4°C), 60 mM NH_4_Cl, 7.5 mM Mg(OAc)_2_, 0.5 mM EDTA, 2 mM DTT). Ribosome concentration in the pellets was measured using *A*_260_ Nanodrop measurements (1 *A*_260_ unit of 70S = 24 pmol 70S). After the second spin, the top 2/3 of the supernatant was collected and transferred into MWCO = 3.5 K dialysis tubing (SnakeSkin) and dialyzed 2 × 1.5 h × 3 l of S150 Extract Buffer at 4°C. For the third dialysis, 3 l of fresh S150 Extract Buffer was used to dialyze overnight (12–15 h). S150 extract was concentrated using Centripreps (3 kDa MWCO) until *A*_260_ = 25 and *A*_280_ = 15. Extract was aliquoted and flash frozen in liquid nitrogen. TP70 was prepared as previously described (26).

### iSAT reactions

5 μl iSAT reactions were performed in 384-well nunc_267461 plates, with four replicates per reaction, and set up as previously described (15,25,37). The Echo 525 Acoustic Liquid Handler was used to aliquot reaction components into the wells. Reaction components were prepared in two separate mixtures: (i) the DNA plasmid with a small amount of premix to enable better liquid handling, and (ii) the remaining reaction components. Premix was mixed with DNA at a volume ratio of 1:2.2 uL premix:DNA so to enable consistent results by increasing viscosity. Reagent mix 2, containing the S150 extract, was added into the wells initially, and then the DNA plasmid mix was aliquoted into each well. Reactions were run in a plate reader at 37°C, measuring fluorescence (excitation: 485 nm, emission: 528 nm) every 15 min and with constant shaking for 15 h. 40% PEG8000 (Sigma-Aldrich P1458-23ML) was added into the reaction premix for a final volume of 10%; 1 M DTT was added at a final volume of 0.2%.

### Plasmid replacement and selection in SQ171fg cells

Electrocompetent *E. coli* SQ171fg cells containing a pCSacB plasmid with kanamycin resistance (KanR) (22,41) were prepared and stored in 50 μl aliquots. The SQ171fg strain is a modified *E. coli* strain that has all seven rRNA operons deleted from the genome. The pCSacB/KanR plasmid carries the sequence for Ribo-Tv2 (43), which serves as the sole rRNA operon in the cell. When pAM552G plasmids carrying the mutated ribosomal operon of interest as well as an ampicillin resistance gene are transformed into the cell, the original pCSacB -Ribo-Tv2 plasmid can be removed by plating on sucrose and carbenicillin (Cb). The success of the selection is then verified by confirming that the strain is no longer resistant to Kan.

50 ng of purified mutant pAM552G plasmid was transformed into 50 μl of cells. Cells were recovered in 850 μl of SOC in a 1.5-ml microcentrifuge tube at 37°C for 1 hour, while shaking at 250 rpm. After 1 hour, 270 μl of the cell recovery was added to 2 ml of Super Optimal broth with Catabolite repression (SOC) containing 50 μg/ml Cb (Cb_50_) and 0.25% sucrose in a 14-ml plastic culture tube. Tubes were incubated at 37°C overnight, for 16–18 h. The tubes were then spun down at room temperature for 5 min at 4000 × g. 2 ml of clear supernatant was removed, leaving the cell pellet to be concentrated into the remaining 270 μl. The concentrated cell suspension was plated on lysogeny broth (LB) agar plates containing 5% sucrose, 50 μg/ml Cb, and 20 μg/ml Ery. Plates were incubated at 37°C until colonies appeared. 8 colonies were picked from each plate and spotted onto two LB-agar plates, one containing Cb_50_ and the other Kan_50_. Colonies that grew successfully on Cb_50_ but not on Kan_50_ were picked and grown overnight in LB with Cb_50_ to be midiprepped using the ZymoPURE™ II Plasmid Midiprep Kit. Midiprepped plasmids were then submitted for Sanger sequencing to confirm the presence of the 23S sequence mutations and ensure that no additional mutations had arisen during the selection process. Constructs that did not yield colonies on the LB-Suc_5%_-Cb_50_-Ery_20_ plates were transformed two subsequent times to ensure that the construct did not support life. Constructs that did not yield ‘clean’ colonies, meaning they grew on both antibiotics, were troubleshot by picking and spot plating additional colonies. If this process was again unsuccessful, transformations were attempted a total of three times before concluding that the construct was not able to support life.

As an additional check that the cells were living solely on the mutated ribosomes, we grew up 5-ml overnight cultures of the successfully transformed SQ171fg strains and purified the total RNA using the Qiagen^TM^ RNeasy Mini kit. We then ran RT-PCRs using the Invitrogen™ SuperScript IV One-Step RT-PCR system to amplify regions of rRNA that were mutated in our variants (primers used listed in Supplementary Table S1 as mutated fragment FP/RP). The products of these RT-PCRs were then submitted for Sanger sequencing.

### Spot growth experiment

SQ171fg strains containing the mutated ribosomes of interest were grown overnight in 3-ml cultures, with Cb_50_. In the morning, the OD_600_ of each culture was measured and normalized to an OD_600_ of 1. Four ten-fold serial dilutions of each construct were prepared (OD = 0.1, 0.01, 0.001, 0.0001). 3-μl of each dilution was carefully pipetted onto a Cb_50_ plate. Plates were incubated at 30°C and 37°C and imaged as soon as a construct at the most dilute concentration showed growth detectable by eye. Spot growth experiments were completed three separate times to ensure consistent results.

### Cloning and selection of randomized Helix 75 library

Primers were designed with randomized nucleotides at the helix 75 library location. Two PCRs were performed using primers with nucleotides randomized at the correct location (Supplementary Table S1). These two fragments were ligated using Gibson assembly and transformed into chemically competent Dh10B cells. The transformation was allowed to recover for one hour at 37°C before being plated on Cb50 and grown overnight at 37°C. Fourteen colonies were picked randomly, and plasmids purified as reported above to test in iSAT.

## RESULTS

Our goal was to establish a high-throughput, computationally guided approach to identify functionally active mutant ribosomes. As model regions to mutate, we focused on helices within the PTC, as the PTC plays the central role in the dynamic process of peptide bond formation. Specifically, we selected Helix 73 (H73), Helix 75 (H75), Helix 91 (H91) and Helix 92 (H92), and combinations thereof. H73 is in the aminoacyl site (A-site) of the PTC and makes contacts with the dynamic ribosomal (r-)protein L3 (44–46). The average conservation of H73 residues explored in this study is 73% across the domains of bacteria and archaea, and three residues (G2046, C2047 and G2621) have > 91% conservation (Supplementary Figure S1). H75 is known to play a role in the assembly of the nascent polypeptide exit tunnel (5) which is essential to proper polypeptide folding; multiple bases in this helix are > 90% conserved (Supplementary Figure S1). Along with helices 76 and 79, H75 forms the base of the L1 stalk (47), which facilitates binding, movement, and release of deacylated tRNAs (48). H91 and H92 together form one side of the highly conserved ‘accommodation corridor,’ where aminoacylated tRNAs are directed into the PTC in a specific orientation. The average conservation value for H91 residues mutated in this work is ∼92%, and the H92 region, which contacts r-protein L14 (46), contains five residues that have greater than 95% conservation (Supplementary Figure S1).

### Establishing stepwise Monte Carlo library selection on Helix 75

**Figure 1.**
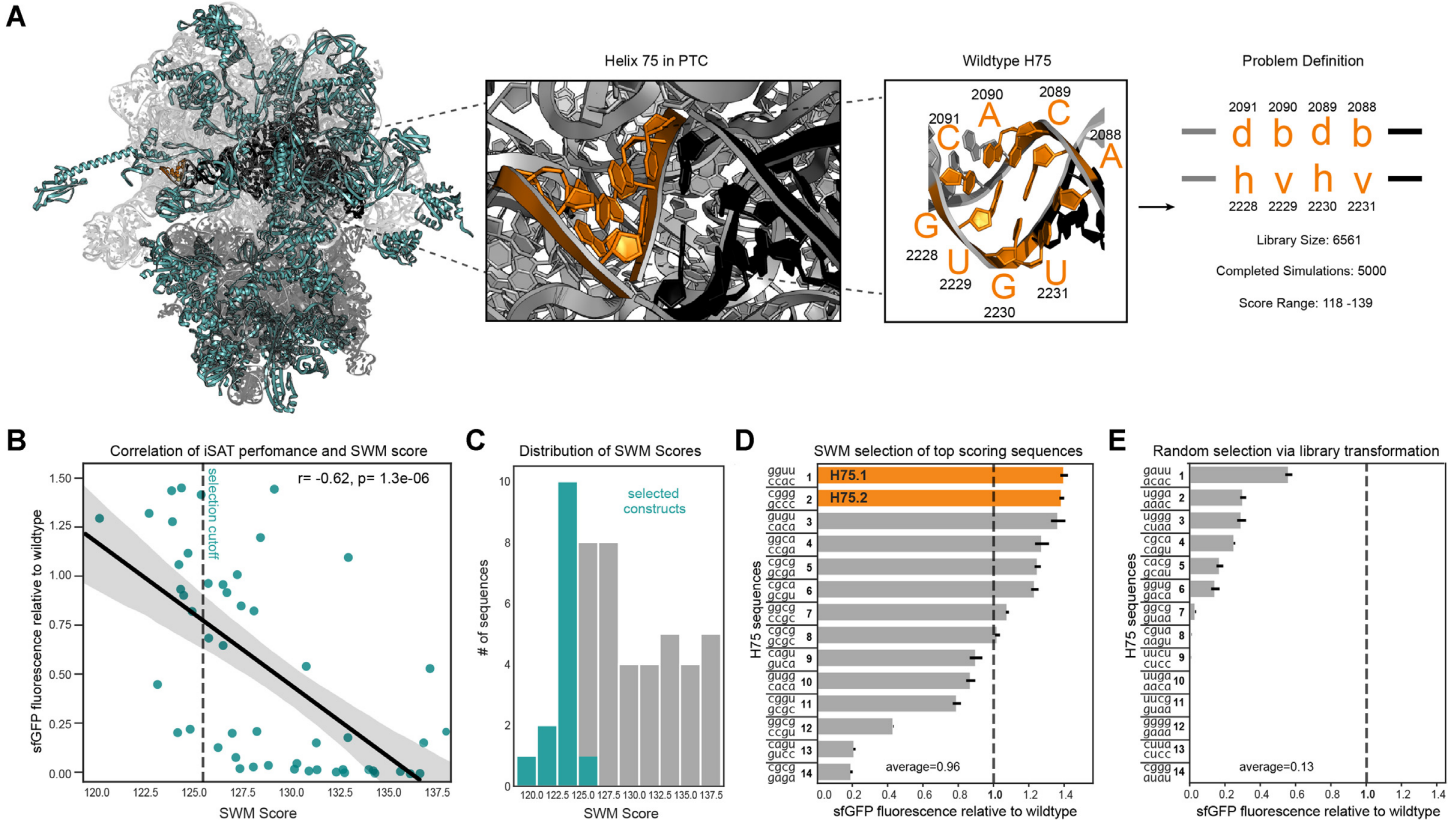
Application of SWM to selection of H75 variants yields high-performing mutants in iSAT. (**A**) Structure and library design for H75. H75, highlighted in orange, sits on the edge of the PTC, which is highlighted in black. Ribosomal proteins are highlighted in teal. Ribosome structure accessed from PDB ID: 4YBB. (**B**) Correlation between iSAT activity of mutants and their SWM scores. Dashed line indicates SWM score cutoff for selection. Gray highlighted region represents the 95% confidence interval. (**C**) Selection scheme for constructs based on their SWM scores. (**D**) Normalized sfGFP expression in iSAT of SWM selected constructs. (**E**) Normalized sfGFP production of randomly selected mutants. Dashed line highlights wild type sfGFP production. Data are presented of means of *n* = 3 experiments with standard deviation shown.

We first established the ability of SWM to computationally design and select mutations in H75 of the 23S rRNA, which sits near the edge of the PTC but has ≥ 90% sequence conservation among all bacterial ribosomes (49). In short, SWM searches libraries via an add-and-delete move method with stochastic sampling and outputs a Rosetta all-atom energy score (34). This score is a linear combination of scaled statistical and physics-based energy terms, which serves as a metric to understand and compare sequence stabilities (50). These simulations and resulting scores account for interactions with nearby residues, whether RNA, protein, or ion. For H75, we created a library of rRNA variants by selecting eight nucleotides that make up the center of the helix and permitted each residue to be anything other than its identity in the wildtype (WT) ribosome (Figure 1A). Using this problem definition, we ran 10 000 SWM design simulations and selected 50 resulting sequences whose scores spanned the energy score range to build (Supplementary Table S2).

With these SWM-scored mutant rRNA sequences at hand, we next tested their performance in the high-throughput iSAT platform, a readout for combined assembly and translational activity. iSAT co-activates the processes of rRNA synthesis and processing, ribosome assembly, and translation in a one-pot *in vitro* reaction (25). Performance in iSAT was quantified by monitoring superfolder green fluorescent protein (sfGFP) expression. The 50 rRNA mutant sequences were tested in iSAT to see if the SWM conformation score was correlated to iSAT performance. Maximum sfGFP synthesized after a 16-hour iSAT reaction incubated at 37°C was measured for each construct and normalized relative to that of the wildtype ribosome control. We observed an inverse correlation (*r* = –0.62, *P* = 1.3e–06) between performance in iSAT and SWM score (Figure 1B). Lower scores, which indicate a more stable rRNA structural conformation, were more likely to yield functional ribosomes in iSAT, a result consistent with our recent work (51). Given this correlation, we moved forward with using SWM score as a metric for selection of successful mutants.

While we initially tested constructs with a broad range of SWM scores to explore the correlation between score and performance in iSAT, we wanted to confirm that using this relationship as a selection criterion would enable identification of highly successful constructs. We therefore picked sequences with scores in the top 30% (Figure 1C, D) and selected an equal number of constructs from a randomized library as a negative control using randomized primers (Figure 1E, Supplementary Table S1) to test in iSAT. Of the constructs selected using our scoring metric, 7/14 of the selected sequences outperformed the WT sequence in iSAT. The average relative performance of SWM selected sequences was 0.96 (Figure 1D) compared to the average of 0.13 for sequences randomly selected from the negative control library (Figure 1E). This indicates that our selection method allows for the identification of variants expressing high levels of sfGFP in iSAT and highlights the flexibility of the PTC to mutations when using the SWM design strategy. Additionally, many of the selected sequences were non-trivial solutions, in that they did not maintain the base pairing pattern of the WT H75. For example, H75.11 (cggu,gcgc), which can have only two Watson-Crick (WC) base pairing interactions as opposed to the four WC base pairs in the WT helix, shows near WT sfGFP levels in iSAT. We also see that many of the selected sequences, such as H75.1, H75.4 and H75.5, do not have a WC interaction at the fourth position between residues 2228 and 2091. Although some crystal structures have found this pair to be closely interacting (39), other studies, specifically mapping secondary structure of the 23S rRNA using base-pairing and stacking interactions (52) show that G2228 is pulled away from C2091 due to a bulging motif at the base of Helix 79. This indicates that our computational modeling approach was able to detect and account for the additional flexibility of this base pair and favorably score sequences that left this region less rigid.

### Application of SWM to select for functional mutated helices in the A and P sites

**Figure 2.**
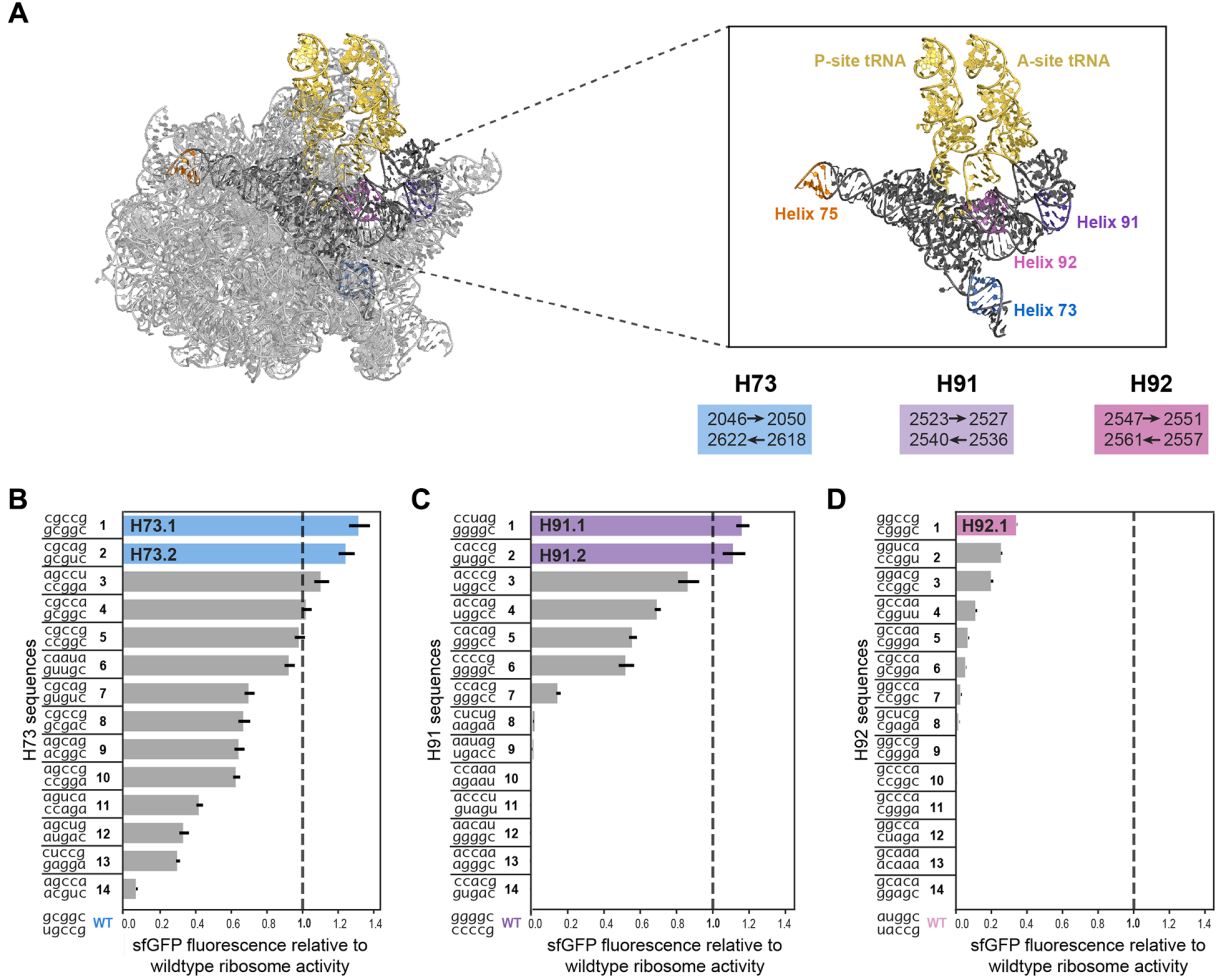
Design of additional helices using SWM yields multiple highly active mutants. (**A**) Structure of the 23S rRNA in gray with PTC highlighted in black. Libraries H73, H75, H91 and H92 shown in blue, orange, purple and pink, respectively. A and P-site tRNAs are highlighted in yellow. Ribosome structure accessed from PDB ID: 7K00. (**B**) iSAT results for selected H73 variants. (**C**) iSAT results for selected H91 variants. (**D**) iSAT results for selected H92 variants. Dashed line indicates the activity of WT, normalized to 1. Data are presented of means of n = 3 experiments with standard deviation shown.

We next sought to use SWM design and selection on additional motifs within the PTC to assess mutational flexibility and find novel ribosomal mutants. We chose H73, H91 and H92, three helices highlighted in Figure 2A (53) that are known to play roles of varying importance to the ribosome's dynamic activity. As previously noted, these three helices are highly sequence conserved (Supplementary Figure S1). Notably, the sequences of H91 are H92 are >90% conserved in the domain of bacteria, and H92 contains three bases that are universally conserved across all domains of life (49). Using a similar approach as for H75, we ran design simulations for H73, H91 and H92, and as above selected 14 sequences from each library that had energy scores in the best 30% of scores to build and test in iSAT (Supplementary Table S3, Supplementary Table S4). All three simulations provided us with functional 23S rRNA sequences, and multiple sequences outperformed the WT control in iSAT (Figure 2B–D). The observed trends highlighted that the ribosomal mutants do not have to exactly mimic WT base pairing patterns to yield high performing variants in iSAT. For example, variants H73.3, H73.4 and H73.5 (Figure 2B), which are all at least as high-performing as WT in iSAT, have at least one base pair that is not a canonical WC base pair. Additionally, our data supported previous findings that variant performance is highly sensitive to even small sequence changes. The identity of even one non-WC base pair can affect sfGFP production considerably; H91.3 and H91.4 are nearly identical, but H91.4 produces 20% less sfGFP due to a single nucleotide change converting a C-C pair to an A-C pair (Figure 2C, Supplementary Table S3). This suggests that 2539C is more favorable than 2539A, perhaps due to interactions occurring between 2539 and nearby rRNA or r-protein residues; or as the result of stronger hydrogen bonding interactions present in a C-C pair than an A-C pair (54). Surprisingly, between H91.3 and H91.6, we observed that changing the first base pair from A-U to C-G and the fourth from C-C to C-G leads to an almost 2-fold knockdown of normalized sfGFP expression. The selected sequences from H92 were the least successful in iSAT, indicating that the ribosome is more sensitive to changes in this helix (Figure 2D). This is likely due to the important role of the post-transcriptionally modified WT base Um2552, which is known to trigger ribosome assembly (55) and whose proper modification by methyltransferase RrmJ may be impaired by mutations to bases in the H92 library. Another factor may be that H92 is specifically recognized by DbpA, an RNA helicase that is known to play an important role in ribosome assembly (56,57), and mutations in H92 affect DpbA’s ability to properly recognize its substrate (6). Despite these design challenges, some of these mutants were functional in iSAT. Of note, we find that canonical base pairing does not guarantee high performance; H92.4 has canonical base pairing but yields less than a third of the sfGFP production of H92.1. A modified library design for H92 that includes additional randomized residues may allow for better compensatory mutations to be identified in this region.

### Combinatorial analysis of top performing sequences highlights complex epistatic interactions in the PTC

**Figure 3.**
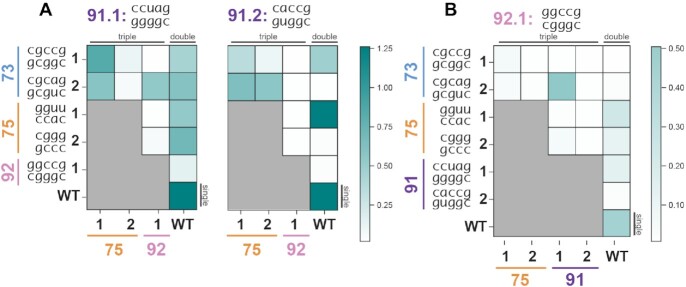
iSAT activities of two and three-way PTC mutant combinations yield constructs with varying sfGFP expression and highlight epistatic interactions. (**A**) H91 mutations kept constant; all constructs shown include a mutated H91, as indicated in the title. (**B**) H92.1 mutant included in all combinations. Data represent the average of *n* = 3 independent experiments.

We next wondered if combining mutations across different helices in the PTC would lead to compensatory, beneficial phenotypes. To test this, we selected top-performing sequences from each library (highlighted in Figures 1D and 2B–D) and constructed all possible combinations of the four library region sequences including WT, yielding a total of 54 combinatorial rRNA constructs to test in iSAT. While none of the constructs with all four library locations mutated produced detectable levels of sfGFP in iSAT, many of the 3-way combinations were highly functional and even competitive with the WT control (Figure 3; Supplementary Table S5, Supplementary Figure S2).

The results uncovered epistatic interactions between helices that show the highly interacting structure and complexity of the PTC. Of note, sfGFP production in iSAT could be recovered by adding additional mutated helices in some cases. For instance, we observed that H91.2 combined with H73.2 (C43) is inactive, but when further combined with either H75.1 or H75.2 (C31, C37) the sfGFP production relative to WT increases to above 43% of WT (Figure 3A, Supplementary Table S5). Although H73/H91 and H75 sit at opposite ends of the PTC, the addition of 8 mutated residues at H75 unexpectedly complements the mutations in H91.2 and H73.2, rendering them compatible. In another example, we showed that the double mutant of H91.2 combined with H73.1 (C20) is moderately functional while H91.2 with H73.2 (C43), which has only one base pair difference from H73.1, does not produce detectable levels of sfGFP. H73’s effects on H91 are different when looking at the H91.1 mutant; H91.1 has 0.50 relative protein production with both H73.1 (C22) and H73.2 (C45). Similarly, while H91.1 is functional as a double mutant with H75.1 (C55) or H75.2 (C60), H91.2 yields very high sfGFP expression when paired with H75.1 (C53) but none with H75.2 (C58) – even though H75.1 and H75.2 perform almost identically in iSAT individually (Figure 1D, Supplementary Table S3). Of note, most triple mutants including H92.1 are inactive except for in C44, its combination with H73.2 and H91.1 (Figure 3B, Supplementary Table S5). In fact, the addition of these 20 mutations from H73.2 and H91.1 endows the ribosome with a greater than 20% increase in normalized sfGFP production compared to any double or single mutant containing H92.1 (Supplementary Table S5), indicating a sensitive relationship between the three helices. This may be explained by role of the L3 protein, which acts as a dynamic switch to coordinate binding of elongation factors and has been reported to interact with helices 73, 91 and 92 as amino-acid charged tRNAs are introduced into the A-site and shuttled to the P-site (44). These mutant combinations highlight four key findings: (i) there exist previously unexplored relationships between helices in the PTC, (ii) considering dynamic and distal interactions in rRNA is essential for successful rRNA library design, (iii) ribosome performance in iSAT is sensitive to even single base pair differences in helices, and (iv) the SWM approach enables large-scale mutations in the PTC despite high sequence conservation.

### Highly mutated, computationally designed ribosomes support life

We then transformed all combinatorial constructs and single-helix mutants into *E. coli* to test whether these mutant ribosomes could support translation of the *E. coli* proteome. We used a previously described selection scheme (15,41). In short, pAM552G plasmids conferring carbenicillin resistance and encoding the mutant ribosomes were individually transformed into the *E. coli* SQ171fg strain (58), which lacks chromosomal rRNA alleles and lives on the pCSacB plasmid carrying the RiboT-v2 sequence (43). The pCSacB plasmid also contains a counter-selectable marker (sacB), which confers sucrose sensitivity, and a kanamycin resistance cassette. Thus, by growing the transformed strains in the presence of carbenicillin and sucrose, the pCSacB plasmid can be eliminated, leaving only the pAM552G ribosome mutant plasmid.

**Figure 4.**
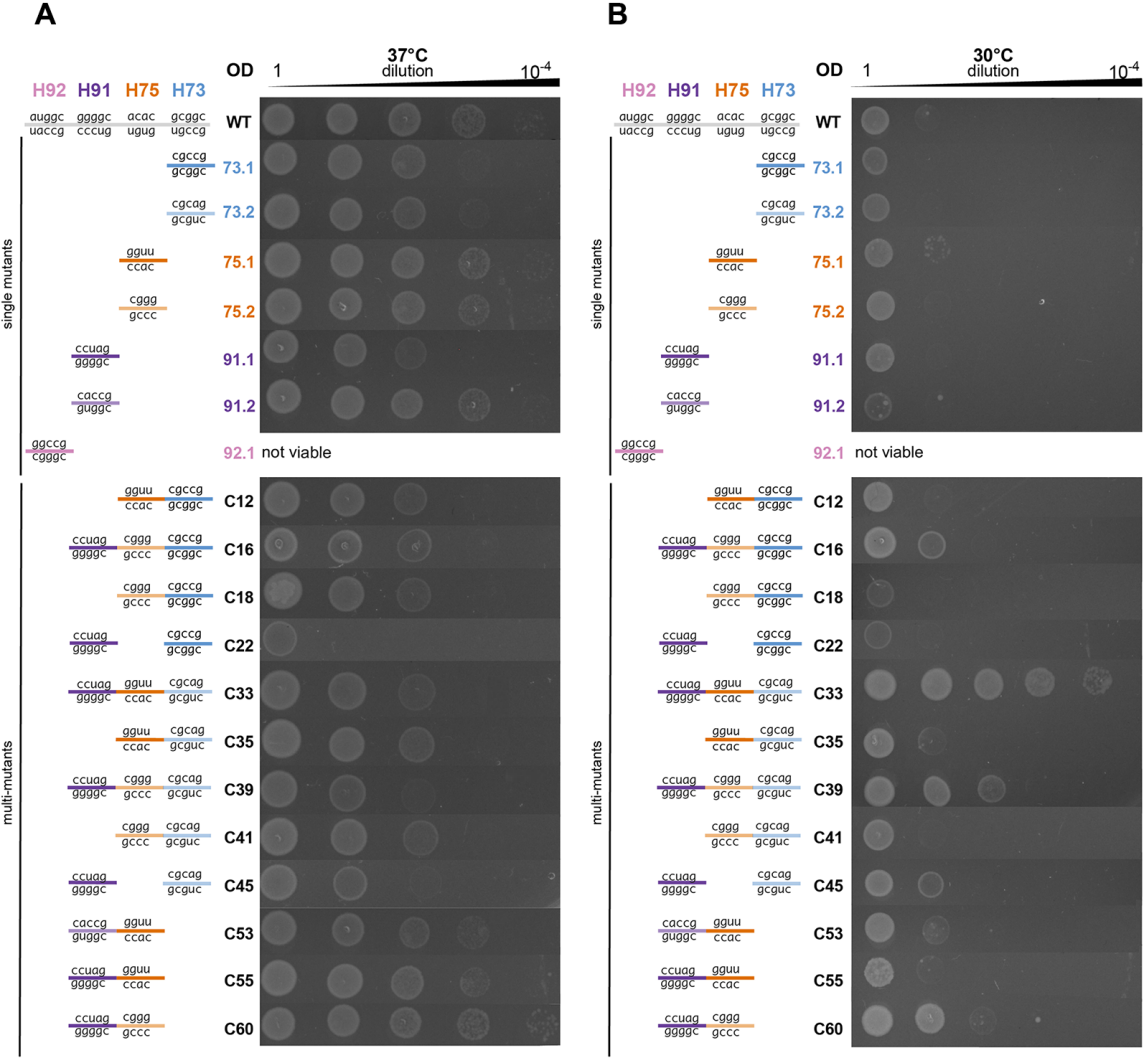
Spot growth assays of controls and combination constructs show that many mutants can support cell growth competitive with wildtype. (**A**) Spot growth assay at 37°C. (**B**) Spot growth assay at 30°C. Data representative of *n* = 3 independent experiments.

While most combinatorial constructs were not able to support life, many of them were successful and enabled cell growth closely resembling that of WT (Figure 4A). Notably, strains C16, C33 and C39, which all harbor ribosomes that have >10% of their PTCs mutated from WT, are still able to support cell survival and growth. Additionally, the *in vivo* analysis highlighted epistatic interactions that differed from those identified in iSAT. For example, we found that strain C45, containing H73.2 and H91.1, grew slowly, but when combined with H75.1 to produce strain C33, grew more robustly. Likewise, the combination of H91.1 with either H75.1 (C55) or H75.2 (C60) exhibited faster growth than of H91.1 alone. Of the constructs that had greater than 1/3 relative sfGFP expression to WT in iSAT (Supplementary Tables S5 and S6), >73% were able to support life; thus, our data suggest performance in iSAT above a certain threshold may serve as a predictor of whether a mutant can support life. However, there were some exceptions. For example, C10 (H73.1-H75.1-H91.1) was high-performing in iSAT but did not support life (Supplementary Table S6). Conversely, strain C39 was able to support cell growth despite its low performance in iSAT. These disparities are likely due to differences in the concentrations of the many dozens of assembly factors involved in ribosome biogenesis in either environment (59). We also measured the growth profiles of these strains at a lower temperature (30°C) to observe any phenotypic changes that may be more pronounced in suboptimal growth conditions (Figure 4B). Surprisingly, strains C33 and C39, which showed slightly impaired growth at 37°C, grew significantly more robustly than WT at the lower temperature. This suggests that the mutations in strains C33 and C39 may lead to improved folding and assembly at lower temperature in cells.

Of note, plasmid exchange in the Squires strain can lead to the appearance of clones that are sucrose resistant and kanamycin sensitive, but still carry the wild type rRNA operon, possibly due to its integration into the genome or plasmid. These clones may consequently express wild type rRNA, convoluting the assessment of whether the mutant ribosomes are supporting life. To ensure that the ribosomes being expressed in our study were comprised of mutant and not WT rRNA, the entire plasmid-borne rRNA operon sequence was confirmed to contain the desired mutations by Sanger sequencing of miniprepped plasmids. The total RNA was then extracted from eight of the combination strains and RT-PCRs of the mutated regions were carried out, confirming that the rRNA being expressed in the cells matched the mutant sequence as well. No wildtype sequences were detected in any of the strains tested, indicating that the cells were harboring and expressing only the mutated ribosomal operon sequence (Supplementary Figure S3).

## DISCUSSION

Here, we developed a computational rRNA structure prediction method to select for highly active ribosomal PTC mutants from complex libraries and explore previously unidentified epistatic interactions between distal helices in the PTC. Our work has several key features.

First, we showed that we can use SWM to successfully select for high performing mutants using an all-atom energy score, and that this approach can serve as a tool to design significantly mutated variants that are not only functional, but even enable higher protein production yields in the cell-free environment than the wildtype ribosome sequence. This finding is consistent with current understanding of the importance of rapid rRNA folding for ribosome assembly and function (60), which has been understood to be a function of the molecule's minimum free energy (61,62). By combining helix mutants, we built functional ribosomes with up to 30 mutations, the most highly mutated designed PTCs to our knowledge, showing that the PTC is amenable to this method of computationally vetted mutation. Many of these multi-mutants were able to support life; some strains showed improved growth phenotypes compared to that of a strain carrying a WT ribosome. Additionally, we observed that constructs that were more successful *in vitro* had a higher probability of being able to support life in cells and identified a general rule for predicting *in vivo* success as a function of iSAT performance, which appears to be agnostic to helix location. By applying this heuristic, future design efforts may be able to test fewer candidates to arrive at successful rRNA sequences.

Second, our approach enabled library design through the lens of three-dimensional structure. We believe this feature is important for rRNA design, as nucleotides that are distant in sequence space are often highly interacting in three-dimensional space such that mutating a single residue can have off-target effects on other rRNA motifs. SWM also allows for unbiased library assessment, which is experimentally challenging due to inherent biases of primer synthesis and template:primer interactions. By computationally investigating large libraries of mutations in the PTC, we were able to explore the folding energy landscape and find alternative minima that retained—and sometimes improved—ribosome function.

Third, select combinatorial mutants in this study highlighted previously unidentified epistatic interactions between helices in the PTC. For example, performance of A-site helix mutants (H91) was strongly affected by mutations in the E-site (H75). This may be attributed to the roles that these helices play in tRNA translocation. H75 forms a three-way helical junction at the base of the L1 stalk, a dynamic feature that travels a path of ∼60 Å to aid in releasing tRNAs from the ribosome (47,63); thus, the sequence of H75 likely affects the bending movement of the stalk. H91, as noted earlier, forms part of the accommodation corridor, which undergoes key conformational changes as the tRNA is moved into the ribosome (64). Mutations in H75 and H91 may therefore be related via the central roles they play in tRNA translocation. Additionally, H92, which interacts closely with the A-site tRNA and also makes up part of the accommodation corridor, is thought to act as a dynamic gate that slows the tRNA acceptor stem before permitting its passage into the P-site (63,65). This activity may be affected by the residue identities of H91, potentially explaining the observed sensitivity of our H92 variants to small changes in H91 sequence. We also found that multi-helix variant performance was highly sensitive to small sequence differences; for example, for mutants carrying both H91.1 and H92.1, a single base pair change in H73 rendered the multi-mutant incapable of producing sfGFP. This may be attributed to the role of H73 in downstream rRNA folding pathways and assembly of the folding tunnel due to its position in the ‘central core’ of the 23S, from which all other domains extend (52). This 23S rRNA core is speculated to fold independently into its active form and create a base for the A and P-sites to form via interactions with r-protein L3 (52). This interaction may thus serve as the basis of epistatic interactions between H73 and H92, as H92 interacts closely with r-protein L14, which forms a tight cluster with L3 and L19 (46,66,67). In addition to the previously discussed Um2552 mutation challenges, changes to H92 also may affect PTC mediated folding of the nascent protein chain as bases A2560 and U2561 have been reported to interact closely with unfolded proteins and play a role in the nucleation of protein folding (68,69). Lastly, while H73 and H91 have been reported to be related via their proximity and interactions with the L3/l14/l19 r-protein cluster (44,66), their relationship with H75, as shown by combinations of H73.1/H91.1 with H75.1/H75.2, has not been previously documented to our knowledge.

Our results suggest that these rRNA motifs are functionally co-dependent, perhaps due to altered mobility of the L1 stalk and tRNA shuttling, PTC-mediated peptide folding, and interactions with r-proteins; and that multi-mutants in these helices, while functional in certain combinations, can render the ribosome inactive if incompatible. Notably, merging highly functional mutants does not guarantee that the resulting variant will be successful; as seen with H75.2 and H91.2 (C58), which individually both outperformed the WT ribosome in iSAT, but when combined abolished iSAT activity (Figures 1D, 2C and 3A). This finding emphasizes the high interconnectivity of the PTC and the need to approach engineering rRNA through a wide lens. It is an oversimplification of the design challenge to identify the most active small-scale mutants to later combine them. Thus, high-throughput screens will likely be required to test diverse mutations in combination to enable discovery of the most promising multi-mutants. The unexpected relationships between distant helices of the PTC underscore the dynamic activity of the ribosomal active site and help improve our understanding of how to account for these kinds of interactions in future ribosome engineering efforts.

Looking forward, we anticipate that energy-based structure predictions such as SWM will be important to facilitate ribosome design. This promises to advance our understanding of rRNA function and molecular translation, as well as accelerate efforts in making modified ribosomes with expanded functions for chemical and synthetic biology.

## DATA AVAILABILITY

Methods and input files used to run SWM simulations are available at https://doi.org/10.5281/zenodo.7230453. Methods and input files used to run sequence conservation analysis are available at https://zenodo.org/badge/latestdoi/523055475. Other data is available in the Supplementary Information.

## Supplementary Material

gkac1036_Supplemental_File
