# Supplementary material for: Computationally-guided design and selection of high performing ribosomal active site mutants
Source: Nucleic Acids Res. 2022 Dec 9;50(22):13143–54. doi: 10.1093/nar/gkac1036 (PMC9825160; doi:10.1093/nar/gkac1036)

Computationally-guided design and selection of high performing ribosomal active site mutants

Camila Kofman[a,b,c], Andrew M. Watkins[d,e], Do Soon Kim[a,b,c], Jessica A. Willi [b,c], Alexandra C. Wooldredge[b,c], Ashty S. Karim[a,b,c], Rhiju Das[d,f], Michael C. Jewett[a,b,c,g]

Affiliations
a. Department of Chemical and Biological Engineering, Northwestern University, Evanston, IL 60208, USA
b. Chemistry of Life Processes Institute, Northwestern University, Evanston, IL 60208, USA
c. Center for Synthetic Biology, Northwestern University, Evanston, IL 60208, USA
d. Department of Biochemistry, Stanford University, Stanford, CA 94305, USA
e. Prescient Design, Genentech, South San Francisco, CA 94080, USA
f. Inceptive Nucleics, Inc. Palo Alto, CA 94304, USA
g. Department of Physics, Stanford University, Stanford, CA 94305, USA
h. Robert H. Lurie Comprehensive Cancer Center and Simpson Querrey Institute, Northwestern University, Chicago, IL 60611, USA

Correspondence

Michael C. Jewett, m-jewett@northwestern.edu

Supplemental Information

Supplementary Tables

Table S1. Primers used in this study.

**H75 Insert FP**	TGAACCTTTACTATAGCTTGBDBDTGAACATTGAGCCTTGATGTGT
**H75 Insert RP**	CCAGTCAAACTACCCACCAGBDBDTGTCCGCAACCCGGATTA
**H75 Backbone FP**	TGGTGGGTAGTTTGACTGGGG
**H75 Backbone RP**	TGGTGGGTAGTTTGACTGGGG
**Mutated fragment FP for RT-PCR (514)**	ACACTCTTTCCCTACACGACGCTCTTCCGATCTcgtaatccgggttgcggac
**Mutated fragment RP for RT-PCR**	GACTGGAGTTCAGACGTGTGCTCTTCCGATCTtcgtactaggagcagcccc

Table S2. iSAT activities, SWM scores, and sequences of constructed H75 mutants. Mutants H75.1-14, highlighted in blue, were selected for by SWM based on the score-cutoff metric as shown in Figure 1B-D.

Name	Normalized iSAT Activity	Standard Deviation	Top SWM Score	Sequence
**H75.1***	1.400	0.022	123.577	gguu, cacc
**H75.2***	1.387	0.013	122.990	cggg,cccg
**H75.3**	1.367	0.041	124.748	gugu,acac
**H75.4**	1.275	0.039	121.649	ggca,agcc
**H75.5**	1.250	0.017	118.696	cgcg,agcg
**H75.6**	1.234	0.021	123.039	cgca,ugcg
**H75.7**	1.079	0.008	123.966	ggcg,cgcc
**H75.8**	1.023	0.014	123.422	cgcg,cgcg
**H75.9**	0.901	0.035	123.527	cagu,acug
**H75.10**	0.871	0.026	123.707	gugg,acac
**H75.11**	0.792	0.023	124.208	cggu,cgcg
**H75.12**	0.433	0.002	122.151	ggcg,ugcc
**H75.13**	0.212	0.006	124.093	cagu,ccug
**H75.14**	0.195	0.007	123.348	cgcg,agag
**H75.15**	1.394	0.028	129.105	guuu,aacc
**H75.16**	1.058	0.010	133.509	uaga,uaua
**H75.17**	0.973	0.006	126.898	acac,gugu
**H75.18**	0.930	0.031	125.170	cggu,cccc
**H75.19**	0.924	0.030	126.049	caac,guug
**H75.20**	0.885	0.005	126.279	gggu,caca
**H75.21**	0.819	0.005	127.129	aggc,acag
**H75.22**	0.794	0.024	127.892	cgcg,cccg
**H75.23**	0.661	0.013	125.199	cgcg,accg
**H75.24**	0.624	0.015	126.054	ggcg,ugac
**H75.25**	0.522	0.008	131.004	gugu,caca
**H75.26**	0.511	0.007	138.373	auag,caca
**H75.27**	0.201	0.006	128.066	cggc,aguc
**H75.28**	0.192	0.005	126.597	cagu,cgug
**H75.29**	0.182	0.027	138.646	gaag,caca
**H75.30**	0.171	0.005	133.484	uacg,ccua
**H75.31**	0.145	0.002	137.995	aucg,caca
**H75.32**	0.145	0.004	131.605	ggcg,uaac
**H75.33**	0.122	0.002	125.756	cggc,acuc
**H75.34**	0.073	0.001	126.812	cggu,cgag
**H75.35**	0.043	0.003	130.445	gagc,ugca
**H75.36**	0.034	0.001	128.751	cgcg,acag
**H75.37**	0.026	0.000	127.855	cgcg,agac
**H75.38**	0.018	0.001	127.055	cagu,cgcg
**H75.39**	0.015	0.000	130.303	cggu,ccac
**H75.40**	0.014	0.001	134.727	ggcg,uaaa
**H75.41**	0.013	0.001	133.192	ugug,acac
**H75.42**	0.012	0.001	131.646	cggu,cgaa
**H75.43**	0.007	0.000	134.992	cggu,caac
**H75.44**	0.006	0.000	131.166	cgcg,acaa
**H75.45**	0.004	0.000	136.609	ccaa,aacc
**H75.46**	0.001	0.000	132.792	cgcg,acac
**H75.47**	-0.002	0.000	133.290	cagu,cgcc
**H75.48**	-0.004	0.000	135.088	guaa,agcg
**H75.49**	-0.006	0.002	137.776	caca,ucac
**H75.50**	-0.007	0.001	137.197	cucg,uccc

Table S3. SWM scores and sequences of H73, H91 and H92 design constructs. Sequences and scores of the 14 constructs selected from each design simulation.

Construct Name	Relative Activity	Standard Deviation	2046-2050	2618-2622	SWM Score
**H73-1**	1.333	0.059	cgccg	cggcg	-50.677
**H73-2**	1.261	0.045	cgcag	cugcg	-54.276
**H73-3**	1.121	0.041	agccu	aggcc	-53.670
**H73-4**	1.037	0.027	cgcca	cggcg	-55.429
**H73-5**	0.998	0.028	cgccg	cggcc	-47.495
**H73-6**	0.941	0.029	caaua	cguug	-40.547
**H73-7**	0.715	0.028	cgcag	cugug	-54.779
**H73-8**	0.686	0.033	cgccg	cagcg	-55.794
**H73-9**	0.659	0.028	agcag	cggca	-38.793
**H73-10**	0.643	0.020	agccg	aggcc	-40.986
**H73-11**	0.435	0.018	aguca	agacc	-48.076
**H73-12**	0.348	0.027	agcug	cagua	-48.787
**H73-13**	0.314	0.011	cuccg	aggag	-48.627
**H73-14**	0.082	0.005	agcca	cugca	-48.101

Construct Name	Relative Activity	Standard Deviation	2523-2527	2536-2540	SWM Score
**H91-1**	1.178	0.036	ccuag	cgggg	-32.745
**H91-2**	1.130	0.062	caccg	cggug	-33.896
**H91-3**	0.880	0.058	acccg	ccggu	-33.291
**H91-4**	0.709	0.016	accag	ccggu	-30.450
**H91-5**	0.573	0.022	cacag	ccggg	-30.475
**H91-6**	0.537	0.042	ccccg	cgggg	-28.604
**H91-7**	0.162	0.012	ccacg	ccggg	-31.576
**H91-8**	0.024	0.002	cucug	aagaa	-32.275
**H91-9**	0.017	0.001	aauag	ccagu	-32.944
**H91-10**	0.010	0.001	ccaaa	uaaga	-30.967
**H91-11**	0.009	0.002	acccu	ugaug	-27.221
**H91-12**	0.008	0.002	aacau	cgggg	-27.104
**H91-13**	0.005	0.001	accaa	cggga	-28.561
**H91-14**	0.005	0.000	ccacg	cagug	-32.170

Construct Name	Relative Activity	Standard Deviation	2547-2551	2557-2561	SWM Score
**H92-1**	0.344	0.002	ggccg	cgggc	-10.061
**H92-2**	0.261	0.003	gguca	uggcc	-8.448
**H92-3**	0.204	0.007	ggacg	cggcc	-6.888
**H92-4**	0.115	0.005	gccaa	uuggc	-6.631
**H92-5**	0.072	0.003	gccaa	agggc	-7.065
**H92-6**	0.058	0.001	cgcca	aggcg	-11.034
**H92-7**	0.031	0.002	ggcca	cggcc	-9.743
**H92-8**	0.022	0.001	gcucg	agagc	-10.205
**H92-9**	0.008	0.000	ggccg	agggc	-8.210
**H92-10**	0.006	0.000	gccca	cggcc	-8.778
**H92-11**	-0.003	0.003	gccca	agggc	-7.389
**H92-12**	-0.003	0.001	ggcca	agauc	-7.392
**H92-13**	-0.004	0.001	gcaaa	aaaca	-7.494
**H92-14**	-0.008	0.000	gcaca	cgagg	-7.942

Table S4. Overall SWM score ranges for each library simulation.

H73 Design Results	
Min Score	-55.794
Max Score	-8.414
Range	64.208

H91 Design Results	
Min Score	-33.896
Max Score	12.121
Range	46.017

H92 Design Results	
Min Score	-11.034
Max Score	15.442
Range	26.476

Table S5. Sequences and iSAT activities of all single and multi-mutants. "Activity" is signal of construct in iSAT relative to the wildtype ribosome's signal.

Name	Mutant Combination	H73 Sequence	H75 Sequence	H91 Sequence	H92 Sequence	Activity	Std. Dev.
**C1**	73.1,75.3,91.2,92.1	cgccg, cggcg	guuu, aacc	caccg, cggug	ggccg, cgggc	-0.003	0.001
**C2**	73.1,75.3,91.2,H92-WT	cgccg, cggcg	guuu, aacc	caccg, cggug	auggc, gccau	0.485	0.007
**C3**	73.1,75.3,91.1,92.1	cgccg, cggcg	guuu, aacc	ccuag, cgggg	ggccg, cgggc	-0.005	0.001
**C4**	73.1,75.3,91.1,H92-WT	cgccg, cggcg	guuu, aacc	ccuag, cgggg	auggc, gccau	0.228	0.066
**C5**	73.1,75.3,H91-WT,92.1	cgccg, cggcg	guuu, aacc	ggggc, guccc	ggccg, cgggc	0.010	0.000
**C6**	73.1,75.3,H91-WT,H92-WT	cgccg, cggcg	guuu, aacc	ggggc, guccc	auggc, gccau	0.397	0.021
**C7**	73.1,75.1,91.2,92.1	cgccg, cggcg	gguu, cacc	caccg, cggug	ggccg, cgggc	-0.001	0.000
**C8**	73.1,75.1,91.2,H92-WT	cgccg, cggcg	gguu, cacc	caccg, cggug	auggc, gccau	0.253	0.030
**C9**	73.1,75.1,91.1,92.1	cgccg, cggcg	gguu, cacc	ccuag, cgggg	ggccg, cgggc	-0.001	0.001
**C10**	73.1,75.1,91.1,H92-WT	cgccg, cggcg	gguu, cacc	ccuag, cgggg	auggc, gccau	0.651	0.033
**C11**	73.1,75.1,H91-WT,92.1	cgccg, cggcg	gguu, cacc	ggggc, guccc	ggccg, cgggc	0.038	0.006
**C12**	73.1,75.1,H91-WT,H92-WT	cgccg, cggcg	gguu, cacc	ggggc, guccc	auggc, gccau	0.854	0.070
**C13**	73.1,75.2,91.2,92.1	cgccg, cggcg	cggg, cccg	caccg, cggug	ggccg, cgggc	-0.004	0.001
**C14**	73.1,75.2,91.2,H92-WT	cgccg, cggcg	cggg, cccg	caccg, cggug	auggc, gccau	0.055	0.016
**C15**	73.1,75.2,91.1,92.1	cgccg, cggcg	cggg, cccg	ccuag, cgggg	ggccg, cgggc	0.004	0.005
**C16**	73.1,75.2,91.1,H92-WT	cgccg, cggcg	cggg, cccg	ccuag, cgggg	auggc, gccau	0.067	0.014
**C17**	73.1,75.2,H91-WT,92.1	cgccg, cggcg	cggg, cccg	ggggc, guccc	ggccg, cgggc	0.002	0.002
**C18**	73.1,75.2,H91-WT,H92-WT	cgccg, cggcg	cggg, cccg	ggggc, guccc	auggc, gccau	0.741	0.057
**C19**	73.1,H75-WT,91.2,92.1	cgccg, cggcg	acac, gugu	caccg, cggug	ggccg, cgggc	-0.003	0.000
**C20**	73.1,H75-WT,91.2,H92-WT	cgccg, cggcg	acac, gugu	caccg, cggug	auggc, gccau	0.350	0.033
**C21**	73.1,H75-WT,91.1,92.1	cgccg, cggcg	acac, gugu	ccuag, cgggg	ggccg, cgggc	-0.006	0.001
**C22**	73.1,H75-WT,91.1,H92-WT	cgccg, cggcg	acac, gugu	ccuag, cgggg	auggc, gccau	0.328	0.046
**C23**	73.1,H75-WT,H91-WT,92.1	cgccg, cggcg	acac, gugu	ggggc, guccc	ggccg, cgggc	0.000	0.003
**C24**	73.2,75.3,91.2,92.1	cgcag, cugcg	guuu, aacc	caccg, cggug	ggccg, cgggc	-0.005	0.001
**C25**	73.2,75.3,91.2,H92-WT	cgcag, cugcg	guuu, aacc	caccg, cggug	auggc, gccau	0.352	0.003
**C26**	73.2,75.3,91.1,92.1	cgcag, cugcg	guuu, aacc	ccuag, cgggg	ggccg, cgggc	-0.003	0.000
**C27**	73.2,75.3,91.1,H92-WT	cgcag, cugcg	guuu, aacc	ccuag, cgggg	auggc, gccau	0.262	0.003
**C28**	73.2,75.3,H91-WT,92.1	cgcag, cugcg	guuu, aacc	ggggc, guccc	ggccg, cgggc	-0.001	0.000
**C29**	73.2,75.3,H91-WT,H92-WT	cgcag, cugcg	guuu, aacc	ggggc, guccc	auggc, gccau	0.845	0.044
**C30**	73.2,75.1,91.2,92.1	cgcag, cugcg	gguu, cacc	caccg, cggug	ggccg, cgggc	-0.001	0.000
**C31**	73.2,75.1,91.2,H92-WT	cgcag, cugcg	gguu, cacc	caccg, cggug	auggc, gccau	0.484	0.027
**C32**	73.2,75.1,91.1,92.1	cgcag, cugcg	gguu, cacc	ccuag, cgggg	ggccg, cgggc	-0.001	0.000
**C33**	73.2,75.1,91.1,H92-WT	cgcag, cugcg	gguu, cacc	ccuag, cgggg	auggc, gccau	0.450	0.019
**C34**	73.2,75.1,H91-WT,92.1	cgcag, cugcg	gguu, cacc	ggggc, guccc	ggccg, cgggc	0.028	0.001
**C35**	73.2,75.1,H91-WT,H92-WT	cgcag, cugcg	gguu, cacc	ggggc, guccc	auggc, gccau	1.243	0.023
**C36**	73.2,75.2,91.2,92.1	cgcag, cugcg	cggg, cccg	caccg, cggug	ggccg, cgggc	0.003	0.001
**C37**	73.2,75.2,91.2,H92-WT	cgcag, cugcg	cggg, cccg	caccg, cggug	auggc, gccau	0.431	0.039
**C38**	73.2,75.2,91.1,92.1	cgcag, cugcg	cggg, cccg	ccuag, cgggg	ggccg, cgggc	0.001	0.001
**C39**	73.2,75.2,91.1,H92-WT	cgcag, cugcg	cggg, cccg	ccuag, cgggg	auggc, gccau	0.035	0.006
**C40**	73.2,75.2,H91-WT,92.1	cgcag, cugcg	cggg, cccg	ggggc, guccc	ggccg, cgggc	0.006	0.002
**C41**	73.2,75.2,H91-WT,H92-WT	cgcag, cugcg	cggg, cccg	ggggc, guccc	auggc, gccau	0.340	0.016
**C42**	73.2,H75-WT,91.2,92.1	cgcag, cugcg	acac, gugu	caccg, cggug	ggccg, cgggc	0.001	0.001
**C43**	73.2,H75-WT,91.2,H92-WT	cgcag, cugcg	acac, gugu	caccg, cggug	auggc, gccau	0.001	0.001
**C44**	73.2,H75-WT,91.1,92.1	cgcag, cugcg	acac, gugu	ccuag, cgggg	ggccg, cgggc	0.419	0.029
**C45**	73.2,H75-WT,91.1,H92-WT	cgcag, cugcg	acac, gugu	ccuag, cgggg	auggc, gccau	0.454	0.051
**C46**	73.2,H75-WT,H91-WT,92.1	cgcag, cugcg	acac, gugu	ggggc, guccc	ggccg, cgggc	0.010	0.002
**C47**	H73-WT,75.3,91.2,92.1	gcggc, gccgu	guuu, aacc	caccg, cggug	ggccg, cgggc	0.000	0.001
**C48**	H73-WT,75.3,91.2,H92-WT	gcggc, gccgu	guuu, aacc	caccg, cggug	auggc, gccau	1.148	0.025
**C49**	H73-WT,75.3,91.1,92.1	gcggc, gccgu	guuu, aacc	ccuag, cgggg	ggccg, cgggc	-0.001	0.000
**C50**	H73-WT,75.3,91.1,H92-WT	gcggc, gccgu	guuu, aacc	ccuag, cgggg	auggc, gccau	0.420	0.026
**C51**	H73-WT,75.3,H91-WT,92.1	gcggc, gccgu	guuu, aacc	ggggc, guccc	ggccg, cgggc	0.051	0.003
**C52**	H73-WT,75.1,91.2,92.1	gcggc, gccgu	gguu, cacc	caccg, cggug	ggccg, cgggc	0.005	0.001
**C53**	H73-WT,75.1,91.2,H92-WT	gcggc, gccgu	gguu, cacc	caccg, cggug	auggc, gccau	1.232	0.006
**C54**	H73-WT,75.1,91.1,92.1	gcggc, gccgu	gguu, cacc	ccuag, cgggg	ggccg, cgggc	0.007	0.002
**C55**	H73-WT,75.1,91.1,H92-WT	gcggc, gccgu	gguu, cacc	ccuag, cgggg	auggc, gccau	0.410	0.022
**C56**	H73-WT,75.1,H91-WT,92.1	gcggc, gccgu	gguu, cacc	ggggc, guccc	ggccg, cgggc	0.179	0.002

**C57**	H73-WT,75.2,91.2,92.1	gcggc, gccgu	cggg, cccg	caccg, cggug	ggccg, cgggc	0.005	0.000
**C58**	H73-WT,75.2,91.2,H92-WT	gcggc, gccgu	cggg, cccg	caccg, cggug	auggc, gccau	0.004	0.005
**C59**	H73-WT,75.2,91.1,92.1	gcggc, gccgu	cggg, cccg	ccuag, cgggg	ggccg, cgggc	0.036	0.003
**C60**	H73-WT,75.2,91.1,H92-WT	gcggc, gccgu	cggg, cccg	ccuag, cgggg	auggc, gccau	0.542	0.023
**C61**	H73-WT,75.2,H91-WT,92.1	gcggc, gccgu	cggg, cccg	ggggc, guccc	ggccg, cgggc	0.089	0.010
**C62**	H73-WT,H75-WT,91.2,92.1	gcggc, gccgu	acac, gugu	caccg, cggug	ggccg, cgggc	0.012	0.000
**C63**	H73-WT,H75-WT,91.1,92.1	gcggc, gccgu	acac, gugu	ccuag, cgggg	ggccg, cgggc	0.101	0.008
**H92.1**	H73-WT,H75-WT,H91-WT,H92-7	gcggc, gccgu	acac, gugu	ggggc, guccc	ggccg, cgggc	0.344	0.002
**H91.1**	H73-WT,H75-WT,H91-11,H92-WT	gcggc, gccgu	acac, gugu	ccuag, cgggg	auggc, gccau	1.178	0.036
**H91.2**	H73-WT,H75-WT,H91-9,H92-WT	gcggc, gccgu	acac, gugu	caccg, cggug	auggc, gccau	1.130	0.062
**H73.1**	H73-10,H75-WT,H91-WT,H92-WT	cgccg, cggcg	acac, gugu	ggggc, guccc	auggc, gccau	1.333	0.059
**H75.2**	H73-WT,H75-39,H91-WT,H92-WT	gcggc, gccgu	cggg, cccg	ggggc, guccc	auggc, gccau	1.387	0.013
**H73.2**	H73-8,H75-WT,H91-WT,H92-WT	cgcag, cugcg	acac, gugu	ggggc, guccc	auggc, gccau	1.261	0.045
**H75.3**	H73-WT,H75-43,H91-WT,H92-WT	gcggc, gccgu	guuu, aacc	ggggc, guccc	auggc, gccau	1.394	0.028
**H75.1**	H73-WT,H75-41,H91-WT,H92-WT	gcggc, gccgu	gguu, cacc	ggggc, guccc	auggc, gccau	1.400	0.022
**Wildtype**	H73-WT,H75-WT,H91-WT,H92-WT	gcggc, gccgu	acac, gugu	ggggc, guccc	auggc, gccau	0.979	0.029

Table S6. Analysis of iSAT data for prediction of ability to support life in cells. Constructs were ranked by their normalized sfGFP yield in iSAT in descending order to visualize trends in iSAT performance and ability to support life. Constructs that were able to support life are highlighted in blue. Of the constructs that had a normalized activity greater than 30% in iSAT, 16/22 (72.7%) were able to support life.

Name	Normalized sfGFP in iSAT	Std. Dev.	Supports life (Y/N)
H75.1	1.400	0.022	Y
H75.2	1.387	0.013	Y
H73.1	1.333	0.059	Y
H73.2	1.261	0.045	Y
C35	1.243	0.023	Y
C53	1.232	0.006	Y
H91.1	1.178	0.036	Y
H91.2	1.130	0.062	Y
C12	0.854	0.070	Y
C18	0.741	0.057	Y
C10	0.651	0.033	N
C60	0.542	0.023	Y
C31	0.484	0.027	N
C45	0.454	0.051	Y
C33	0.450	0.019	Y
C37	0.431	0.039	N
C44	0.419	0.029	N
C55	0.410	0.022	Y
C20	0.350	0.033	N
H92.1	0.344	0.002	N
C41	0.340	0.016	Y
C22	0.328	0.046	Y
C8	0.253	0.030	N
C4	0.228	0.066	N
C56	0.179	0.002	N
C63	0.101	0.008	N
C61	0.089	0.010	N
C16	0.067	0.014	Y
C14	0.055	0.016	N
C51	0.051	0.003	N
C11	0.038	0.006	N
C59	0.036	0.003	N
C39	0.035	0.006	Y
C34	0.028	0.001	N
C62	0.012	0.000	N
C5	0.010	0.000	N
C46	0.010	0.002	N
C54	0.007	0.002	N
C40	0.006	0.002	N
C52	0.005	0.001	N
C57	0.005	0.000	N
C15	0.004	0.005	N
C58	0.004	0.005	N
C36	0.003	0.001	N
C17	0.002	0.002	N
C38	0.001	0.001	N
C42	0.001	0.001	N
C43	0.001	0.001	N
C23	0.000	0.003	Y
C47	0.000	0.001	N
C7	-0.001	0.000	N
C9	-0.001	0.001	N
C28	-0.001	0.000	N
C30	-0.001	0.000	N
C32	-0.001	0.000	N
C49	-0.001	0.000	N
C1	-0.003	0.001	N
C19	-0.003	0.000	N
C26	-0.003	0.000	N
C13	-0.004	0.001	N
C3	-0.005	0.001	N
C24	-0.005	0.001	N
C21	-0.006	0.001	N

Supplementary Figures

Figure S1. 23S rRNA sequence alignments for regions mutated in this study. 1,614 bacterial and archaeal 23S rRNA sequences were aligned to visualize the sequence conservation in the helices explored in this study.

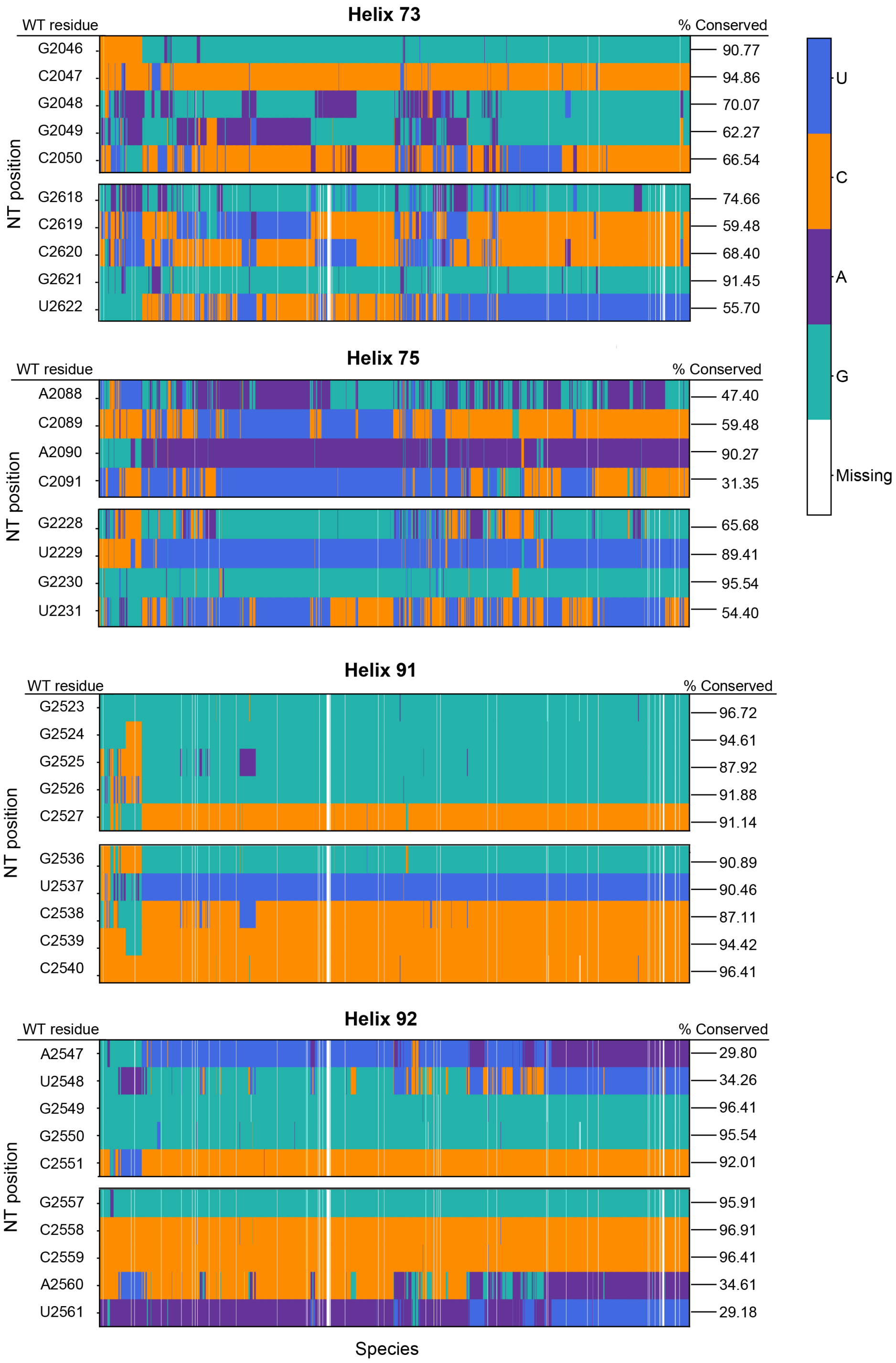

Figure S2. Complete heatmaps of single, double and triple combination construct activities in iSAT. Heatmaps show expression of sfGFP in iSAT normalized by the amount produced by the wildtype control sequence.

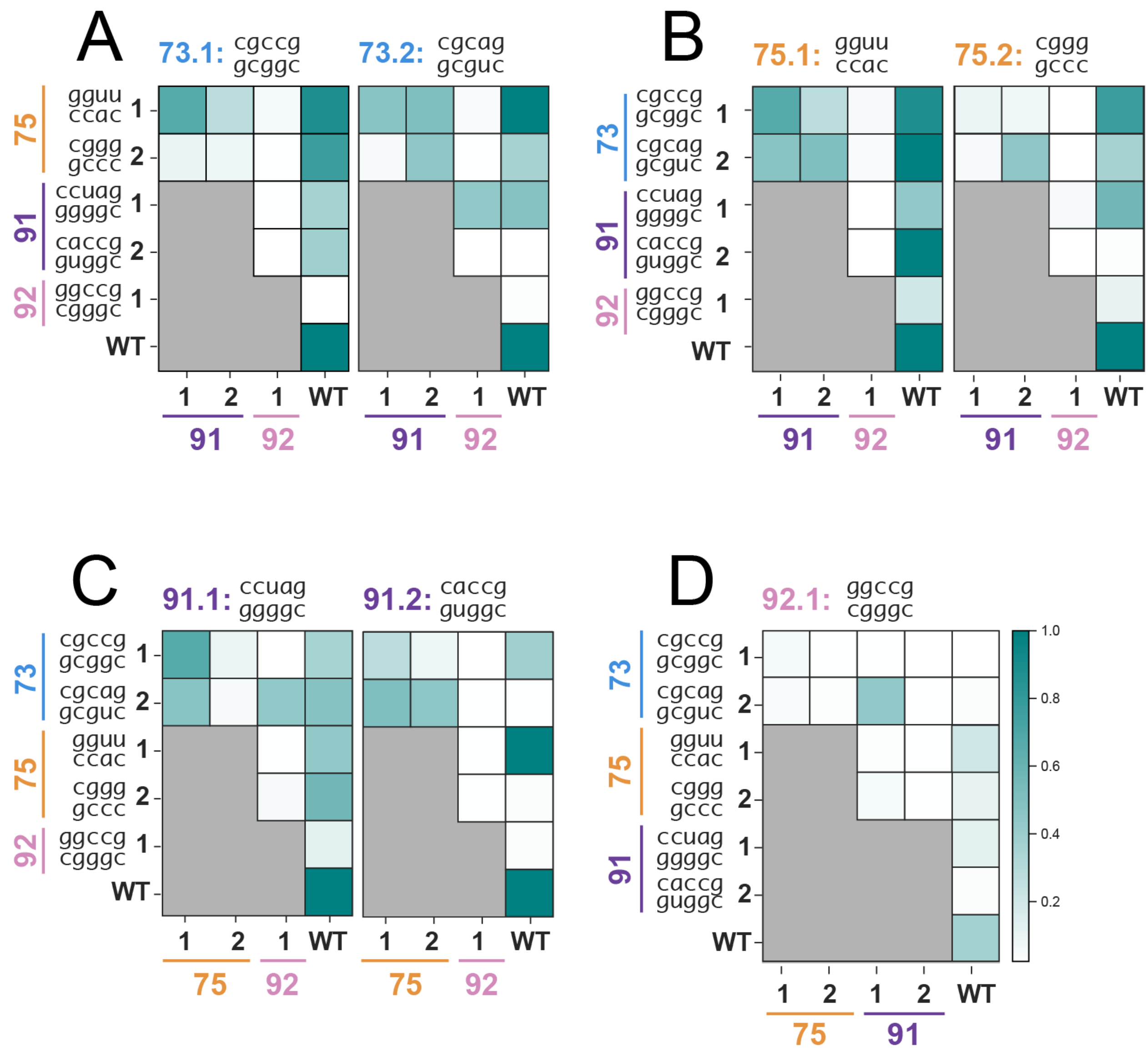

Figure S3. Alignment of Sanger sequencing of RT-PCR products from total RNA extraction of SQ171fg strains carrying mutant ribosomes after selection. Alignment performed using SnapGene (from Insightful Science; available at snapgene.com). Mutations match expected genotypes as shown in Table S5.

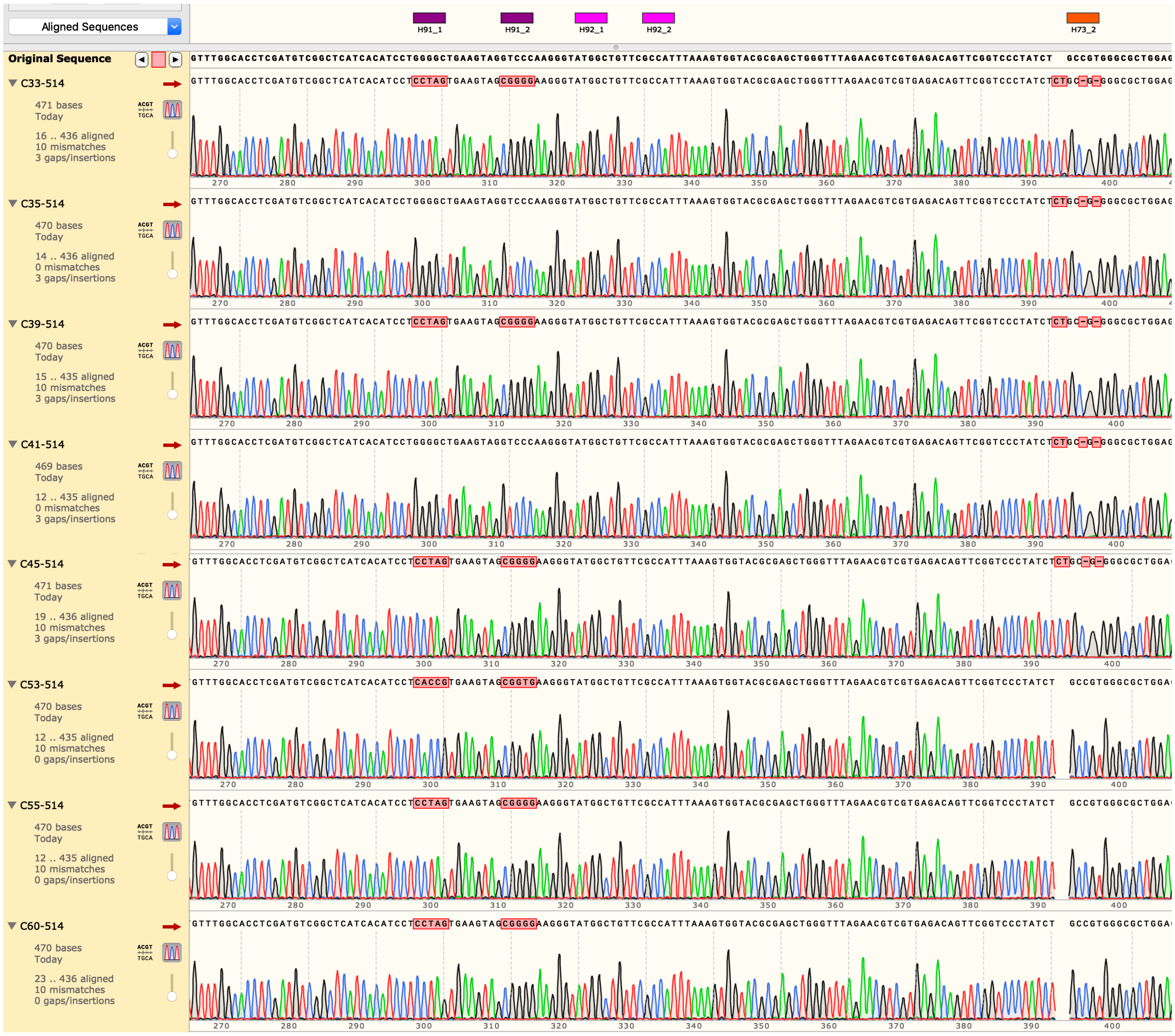

Supplement: gkac1036_Supplemental_File [file gkac1036_supplemental_file.pdf]
